# Pathological Features and Differential Efficacy of Cisplatin-Based Adjuvant Chemotherapy in Lung Cancer Harboring Epidermal Growth Factor Receptor Mutations

**DOI:** 10.5761/atcs.oa.24-00149

**Published:** 2025-01-21

**Authors:** Takafumi Kabuto, Toshi Menju, Shigeto Nishikawa, Kazuhiro Terada, Akihiko Yoshizawa, Hiroshi Date

**Affiliations:** 1 Department of Thoracic Surgery, Kyoto University Hospital, Kyoto, Kyoto, Japan; 2 Department of Thoracic Surgery, Japanese Red Cross Wakayama Medical Center, Wakayama, Wakayama, Japan; 3 Department of Diagnostic Pathology, Toyooka Public Hospital, Toyooka, Hyogo, Japan; 4 Department of Diagnostic Pathology, Kyoto University Hospital, Kyoto, Kyoto, Japan; 5 Department of Diagnostic Pathology, Nara Medical University, Kashihara, Nara, Japan

**Keywords:** lung cancer, surgery, EGFR mutation, EGFR-TKI, adjuvant chemotherapy

## Abstract

**Purpose:** We aimed to elucidate the efficacy of conventional cisplatin-based adjuvant chemotherapy for patients with lung cancers harboring epidermal growth factor receptor (EGFR) mutation.

**Methods:** This retrospective cohort study included 110 patients (EGFR mutation group: n = 51; EGFR wild-type group: n = 59) receiving cisplatin-based adjuvant chemotherapy following complete resection of non-small-cell non-squamous-cell lung cancer (2010–2021). Clinicopathological characteristics, recurrence-free survival (RFS), and overall survival (OS) were investigated.

**Results:** The pStage distribution was not statistically different. The EGFR mutation group was characterized by more advanced pN, papillary predominance, and presence of micropapillary components, whereas the EGFR wild-type group exhibited more advanced pT and solid predominant patterns. The median RFS was significantly worse in the EGFR mutation group (23.0 vs. 76.1 months, *p* = 0.017). Nevertheless, the median OS was not significantly different (85.6 months vs. not reached, *p* = 0.151). Multivariable analysis demonstrated that EGFR mutation and lymphatic invasion were significant risk factors in RFS; however, no independent factors were identified in OS.

**Conclusions:** Cisplatin-based adjuvant chemotherapy might be less effective in patients with EGFR-mutated lung cancer. The style of progression and histological pattern related with EGFR mutation may be associated with the efficacy of adjuvant chemotherapy and poor RFS.

## Introduction

Lung cancer remains one of the leading causes of cancer-related death worldwide.^1,2)^ Surgical resection is an initial treatment choice for early-stage lung cancer. Adjuvant chemotherapy is recommended for patients with advanced lung cancer due to the common recurrence of disease.^3–5)^ Currently, a cisplatin-based platinum-doublet regimen is the recommended option for adjuvant chemotherapy. This recommendation has not changed since the late 2000s.^4–6)^ In patients with lung cancer harboring epidermal growth factor receptor mutation (EGFRmt), adjuvant therapy with the first-generation EGFR-tyrosine kinase inhibitor (EGFR-TKI) was not associated with a survival benefit.^7,8)^ However, the ADAURA trial recently showed the significant effectiveness of adjuvant therapy with osimertinib for patients with lung cancer harboring EGFR mutation.^9)^ The high effectiveness led to an improvement in disease-free survival and overall survival (OS).^10)^ The impressive result altered the standard strategy of adjuvant therapy. Thus, postoperative treatment for lung cancer with EGFRmt is gaining attention. However, the efficacy of conventional cisplatin-based adjuvant chemotherapy for those patients and the clinicopathological relationship between histological subtypes and EGFR status among the patients receiving adjuvant chemotherapy are unclear. Reviewing the outcomes of conventional therapy is important in deciding to recommend adjuvant therapy with osimertinib.

The aim of this study was to elucidate the efficacy of postoperative cisplatin-based chemotherapy for non-small-cell non-squamous cell lung cancer with EGFRmt and investigate the difference of clinicopathological characteristics.

## Materials and Methods

### Patients

We retrospectively reviewed the patients with non-small-cell non-squamous cell lung cancer, who had undergone complete resection followed by cisplatin-based adjuvant chemotherapy at Kyoto University Hospital from 2010 to 2021. EGFRmt was routinely tested using the resected specimen obtained during the initial surgery. The patients were classified into the EGFRmt and EGFR wild-type (EGFRwt) groups. The prognosis of each EGFRmt (Exons 18, 19, and 21) was also analyzed. In this study, the histological diagnosis and pathological stage were revised by two pulmonary pathologists according to the 8th edition of TNM classification.

Patients who underwent adjuvant chemotherapy other than a cisplatin-based regimen (oral tegafur-uracil therapy, carboplatin-based regimen, and EGFR-TKI), induction chemotherapy or chemoradiotherapy, salvage surgery after systemic chemotherapy, or definitive chemoradiotherapy for initially unresectable lung cancer were excluded from the study.

The endpoints were the clinicopathological characteristics, recurrence-free survival (RFS), and OS. RFS were defined as the period of time from surgery to the date of death by any cause or clinical diagnosis of primary tumor recurrence. OS was defined as the period of time from surgery to the date of death by any cause. The data cutoff date for this analysis was July 21, 2023. Patients who were lost to follow-up without any events were censored at the last known date of follow-up.

### Statistical analysis

Differences between groups with respect to normally and non-normally distributed continuous variables were assessed using the Student’s *t*-test and Mann–Whitney *U* test as appropriate. Categorical variables were analyzed using the chi-squared test or Fisher’s exact test, as appropriate. The Kaplan–Meier method and log-rank tests were used to draw curves for RFS and OS. Cox proportional hazards analysis was performed by selecting recurrence factors reported in previous studies. All *p* value were two-sided, and *p* <0.05 denoted statistical significance. All statistical analyses were performed using the JMP Pro software (version 17; SAS Institute Inc., Cary, NC, USA).

## Results

**Fig. 1:**
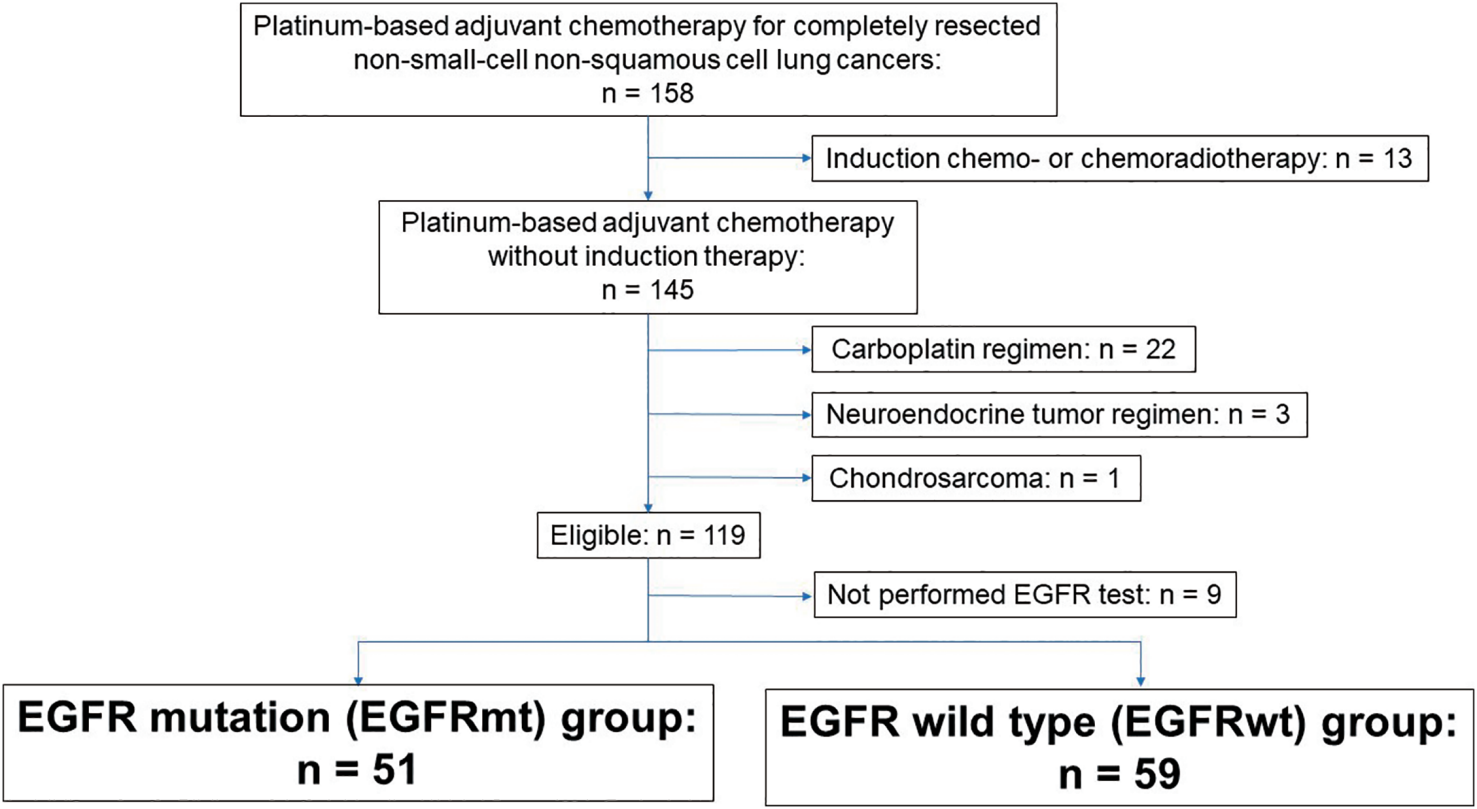
Patient flow. EGFR: epidermal growth factor receptor; EGFRmt: EGFR mutation; EGFRwt: EGFR wild-type

We identified 120 patients who met the inclusion criteria (**Fig. 1**). EGFR tests were performed in 110 patients, who were analyzed in this study.

The clinicopathological characteristics of patients are summarized in **Table 1**. The EGFRmt group had more female and never-smoker patients than the EGFRwt group as previously reported.^2)^ The median age and observation period were not statistically different between the groups. Regarding resection, the EGFRmt group had more patients who underwent simple lobectomy, while only the EGFRwt group included patients who underwent lobectomy with chest wall resection; however, these differences were not statistically significant. Regarding the pathological stage, there was no significant difference, with almost an equal distribution observed between the two groups. There was one case in each group of patients who were considered eligible for adjuvant chemotherapy based on the staging at the time due to tumor size, but were judged to be pathological stage I in the 8th edition of the TNM classification. The EGFRmt group had more advanced pN stage, papillary predominance and micropapillary components, while the EGFRwt group was characterized by more advanced pT stage and solid pattern predominance.

**Table 1:** Patient clinicopathological characteristics

Characteristic		Total (*n* = 110)	EGFRmt group (*n* = 51)	EGFRwt group (*n* = 59)	*p* value
Age (years)	Median	65 (38–76)	64 (40–76)	66 (38–75)	0.754
Sex	Male	50 (45.5%)	16 (31.4%)	34 (57.6%)	0.006
	Female	60 (54.6%)	35 (68.6%)	25 (42.4%)	
Follow-up (months)	Median	56.8 (6.8–164.4)	58.8 (7.7–162.3)	50.8 (6.8–164.4)	0.412
Smoking history	No	49 (44.6%)	34 (66.7%)	15 (25.4%)	<0.001
	Yes	61 (55.5%)	17 (33.3%)	44 (74.6%)	
Resection	Lobectomy	95 (86.4%)	47 (92.2%)	48 (81.4%)	0.176
	Bilobectomy	1 (0.9%)	1 (2.0%)	0 (0%)	
	Segmentectomy	1 (0.9%)	0 (0%)	1 (1.7%)	
	Lobectomy with chest wall resection	4 (3.6%)	0 (0%)	4 (6.8%)	
	Sleeve lobectomy	8 (7.3%)	3 (5.9%)	5 (8.5%)	
	Pneumonectomy	1 (0.9%)	0 (0%)	1 (1.7%)	
Cisplatin cycle	1–2	25 (22.7%)	9 (17.7%)	16 (27.1%)	0.263
	3–4	85 (77.3%)	42 (82.4%)	43 (72.9%)	
Histology	Adenocarcinoma	100 (90.9%)	48 (94.1%)	52 (88.1%)	0.334
	Others (adenosquamous cell carcionoma, pleomorphic carcinoma)	10 (9.1%)	3 (5.9%)	7 (11.9%)	
pStage	I	2 (1.8%)	1 (2.0%)	1 (1.7%)	0.872
	IIB	52 (47.3%)	22 (43.1%)	30 (50.9%)	
	IIIA	46 (41.8%)	23 (45.1%)	23 (39.0%)	
	IIIB	10 (9.1%)	5 (9.8%)	5 (8.5%)	
pT	1	37 (33.6%)	21 (41.2%)	16 (27.1%)	0.005
	2	35 (31.8%)	21 (41.2%)	14 (23.7%)	
	3	30 (27.3%)	8 (15.7%)	22 (37.3%)	
	4	8 (7.3%)	1 (2.0%)	7 (11.9%)	
pN	0	27 (24.6%)	4 (7.8%)	23 (39.0%)	<0.001
	1	38 (34.6%)	20 (39.2%)	18 (30.5%)	
	2	45 (40.9%)	27 (52.9%)	18 (30.5%)	
Pleural invasion	0	64 (58.2%)	30 (58.8%)	34 (57.6%)	0.041
	1	28 (25.5%)	16 (31.4%)	12 (20.3%)	
	2	7 (6.4%)	4 (7.8%)	3 (5.1%)	
	3	11 (10.0%)	1 (2.0%)	10 (17.0%)	
Lymphatic invasion	Absence	80 (72.7%)	37 (72.6%)	43 (72.9%)	0.969
	Presence	30 (27.3%)	14 (27.5%)	16 (27.1%)	
Vascular invasion	Absence	54 (49.1%)	24 (47.1%)	30 (50.9%)	0.692
	Presence	56 (50.9%)	27 (52.9%)	29 (49.2%)	
Pulmonary metastasis	Absence	97 (88.2%)	46 (90.2%)	51 (86.4%)	0.572
	Presence	13 (11.8%)	5 (9.8%)	8 (13.6%)	
Histological predominance	Lepidic	4 (3.6%)	2 (3.9%)	2 (3.4%)	<0.001
	Acinar	17 (15.5%)	11 (21.6%)	6 (10.2%)	
	Papillary	40 (36.4%)	26 (51.0%)	14 (23.7%)	
	Solid	28 (25.5%)	6 (11.8%)	22 (37.3%)	
	Micropapillary	8 (7.3%)	4 (7.8%)	4 (6.8%)	
	Others	13 (11.8%)	2 (3.9%)	11 (18.6%)	
Micropapillary patterns	Absence	40 (36.4%)	9 (17.7%)	31 (52.5%)	<0.001
	Presence	70 (63.6%)	42 (82.4%)	28 (47.5%)	
Solid patterns	Absence	48 (43.6%)	25 (49.0%)	23 (39.0%)	0.438
	Presence	62 (56.4%)	26 (51.0%)	36 (61.0%)	
EGFR mutation	Exon 18		2 (3.9%)		
	Exon 19		30 (58.8%)		
	Exon 21		19 (37.3%)		

EGFR: epidermal growth factor receptor; EGFRmt: EGFR mutation; EGFRwt: EGFR wild type

**Fig. 2:**
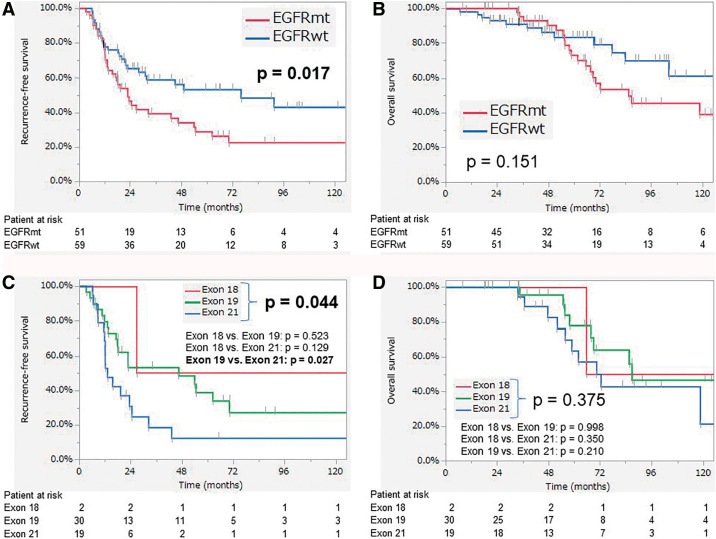
(A) Recurrence-free survival and (B) overall survival (red line: EGFRwt group, blue line: EGFRmt group). (C) Recurrence-free survival of EGFRmt patterns and (D) overall survival of EGFRmt patterns. EGFR: epidermal growth factor receptor; EGFRmt: EGFR mutation; EGFRwt: EGFR wild-type

RFS was significantly worse in the EGFRmt group than the EGFRwt group (*p* = 0.017) (**Fig. 2A**). The 3- and 5-yr recurrence-free rate were 39.0% and 28.7% in EGFRmt group and 58.6% and 53.0% in the EGFRwt group, respectively. There were no significant differences in OS (*p* = 0.151, **Fig. 2B**). The prognoses of all EGFRmt types are also shown in **Figs. 2C** and **2D**. There was a significant difference in RFS among the EGFRmt types (*p* = 0.044), and the Exon 19 group exhibited better RFS than the Exon 21 group (*p* = 0.027). There were no statistically differences in OS among the EGFRmt types.

**Fig. 3:**
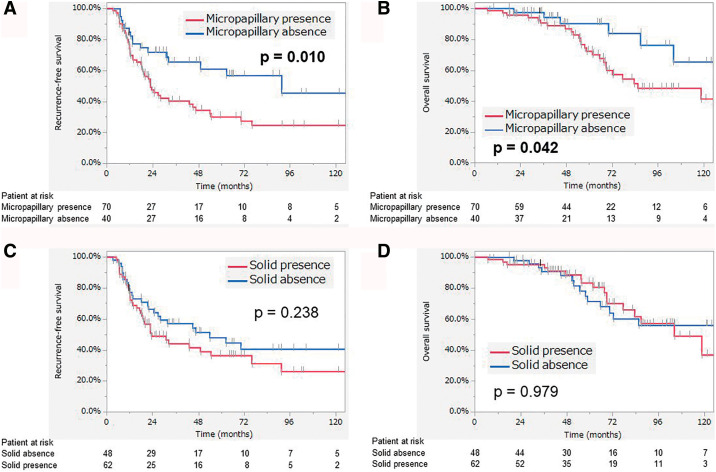
Results of subgroup analysis. (A) Recurrence-free survival in patients with micropapillary pattern presence, (B) overall survival in patients with micropapillary pattern presence, (C) recurrence-free survival in patients with solid pattern presence, and (D) overall survival in patients with solid pattern presence. The red and blue lines indicate the presence and absence of a histological pattern, respectively.

**Fig. 4:**
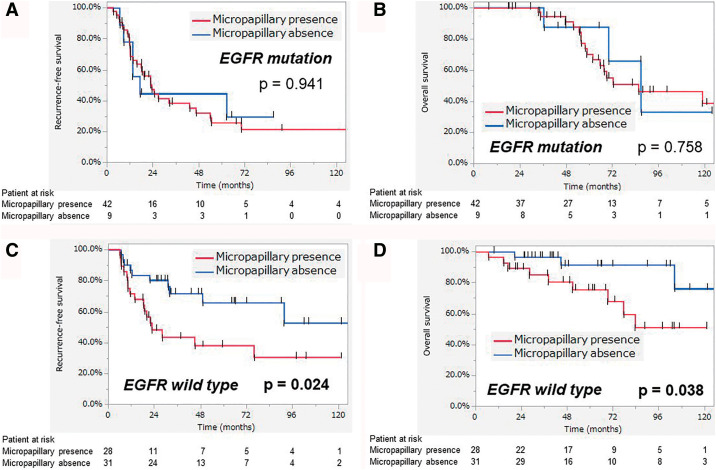
(A) Recurrence-free survival of patients with micropapillary pattern presence in the EGFRmt group and (B) overall survival of patients with micropapillary pattern presence in the EGFRmt group. (C) Recurrence-free survival of patients with micropapillary pattern presence in the EGFRwt group and (D) overall survival of patients with micropapillary pattern presence in the EGFRwt group. The red and blue lines indicate the presence and absence of a histological pattern, respectively. EGFR: epidermal growth factor receptor; EGFRmt: EGFR mutation; EGFRwt: EGFR wild-type.

We also performed a subgroup analysis by dividing the population according to the presence of micropapillary components and a solid pattern (**Fig. 3**). Patients with micropapillary components had a significantly worse prognosis in terms of both RFS (*p* = 0.010) and OS (*p* = 0.042) compared with the other patients. However, the importance of micropapillary components as a prognostic factor depended on the EGFR status (**Fig. 4**). Although differences in RFS and OS were maintained in the EGFRwt group, there were no significant differences observed in the EGFRmt group. In contrast, the presence of a solid pattern was not linked to any statistically significant differences in prognosis.

The primary recurrence pattern recorded during the observation period and the first line treatment administered at the time of recurrence was summarized in **Table 2**. The recurrence rates were 70.6% and 42.4% in the EGFRmt group and EGFRwt group, respectively. The primary recurrence organs were not significantly different between the two groups.

**Table 2:** First recurrence site and the first line treatment

		All (*n* = 110)	EGFRmt group (*n* = 51)	EGFRwt group (*n* = 59)	*p* value
Recurrence		61 (55.5%)	36 (70.6%)	25 (42.4%)	0.004
First recurrence site (overlapping)	Thoracic area	54	30	24	
	Extrathoracic area	35	21	14	
First line treatment	EGFR-TKI	30 (49.2%)	30 (83.3%, the first- and second-generation: *n* = 19, osimertinib: *n* = 11)	0 (0%)	<0.001
	Systemic chemotherapy (cytotoxic chemotherapy and/or immunotherapy)	12 (19.7%)	0 (0%)	12 (48.0%)	
	CRT (+ durvalumab)	9 (14.8%)	2 (5.6%)	7 (28.0%)	
	Surgery	3 (4.9%)	0 (0%)	3 (12.0%)	
	Radiotherapy	4 (6.6%)	3 (8.3%)	1 (4.0%)	
	Observation	1 (1.6%)	0 (0%)	1 (4.0%)	
	Unknown	2 (3.3%)	1 (2.8%)	1 (4.0%)	
Death		32 (29.1%)	20 (39.2%)	12 (20.3%)	0.036
Death cause	Lung cancer	28 (87.5%)	18 (90.0%)	10 (83.3%)	0.715
	Others	3 (9.4%)	2 (10.0%, arrythmia: *n* = 1, suffocation: *n* = 1)	1 (8.3%, pancreatic cancer)	
	Unknown	1 (3.1%)	0 (0%)	1 (8.3%)	

EGFR: epidermal growth factor receptor; EGFRmt: EGFR mutation; EGFRwt: EGFR wild-type; EGFR-TKI: EGFR-tyrosine kinase inhibitor; CRT: chemoradiotherapy

EGFR-TKI was used as the first treatment for 83.3% of patients in the EGFRmt group. The other patients in the EGFRmt group underwent local interventions, such as radiotherapy or chemoradiotherapy. In the EGFRwt group, 48.0% of the patients with recurrence received systemic therapy and 44.0% underwent local therapy (chemoradiotherapy, surgery, and radiotherapy). Lung cancer was the primary cause of death during the observation period (87.5%).

The result of the Cox proportional hazards analysis was shown in **Table 3**. Regarding RFS, EGFRmt (hazard ratio [HR]: 1.79, 95% confidential interval [CI]: 1.02–3.15, *p* = 0.041) and lymphatic invasion (HR: 2.60, 95% CI: 1.49–4.53, *p* <0.001) were identified as significant risk factors. There were no significant risks factors identified for OS.

**Table 3:** Cox proportional hazard analysis

	RFS			OS		
	HR	95% CI	*p* value	HR	95% CI	*p* value
pStage > II	1.68	1.00–2.83	0.052	1.84	0.85–3.98	0.123
EGFR mutation	1.79	1.02–3.15	0.041	1.39	0.65–3.00	0.398
Lymphatic invasion	2.60	1.49–4.53	<0.001	2.21	1.00–4.91	0.051
Vascular invasion	1.16	0.65–2.09	0.616	1.32	0.57–3.06	0.520
Micropapillary	1.57	0.84–2.91	0.157	1.64	0.63–4.28	0.311
Solid	1.40	0.79–2.49	0.246	0.94	0.43–2.05	0.878

RFS: recurrence-free survival; OS: overall survival; HR: hazard ratio; CI: confidence interval; pStage: pathological stage; EGFR: epidermal growth factor receptor

## Discussion

This study showed a similar distribution of pathological stages and a direct comparison between EGFRmt and EGFRwt in patients receiving cisplatin-based adjuvant chemotherapy. We could assess the effect of cisplatin-based adjuvant chemotherapy because the data were collected before the approval of adjuvant molecular-targeted and immune checkpoint inhibitor therapy. The results demonstrated the lower efficacy of cisplatin-based adjuvant chemotherapy and the clear difference in RFS for patients with lung cancer harboring EGFRmt versus those without mutations. In addition, we identified very interesting and different pathological characteristics according to EGFR status, such as the style of progression and the histological patterns. Several studies have discussed the relationship between EGFR status and platinum-based adjuvant therapy; however, they have compared patients with and without adjuvant chemotherapy for each EGFR status in retrospective analyses, which had a limitation of an unavoidable selection bias in that the adjuvant chemotherapy group had patients with a good performance status, fewer comorbidities, and fewer postoperative complications who would endure the adjuvant chemotherapy, whereas the non-adjuvant chemotherapy group had the opposite population.^11,12)^ To the best of our knowledge, this is the first study to analyze real-world data of adjuvant chemotherapy for lung cancer with and without EGFR mutations directly compared under the same conditions in addition to detailed clinicopathological information according to the 8th TNM classification.

RFS has been a surrogate primary endpoint for survival in the major clinical trials to evaluate biological response to treatment, because OS reflects the effects of the treatment after progression, such as an EGFR-TKI, and the deaths from other diseases except lung cancer.^13)^ In the subgroup analysis of JIPANG study, Takahashi et al. also showed worse RFS in the patients with lung cancer harboring EGFRmt than in those with EGFRwt lung cancer. This analysis prospectively compared cisplatin plus vinorelbine versus cisplatin plus pemetrexed as adjuvant chemotherapy for the patients with completely resected lung cancer.^14)^ Another study also reported that the prognosis of lung cancer with EGFRmt might be worse than EGFRwt lung cancer in the adjuvant setting.^15,16)^ Moreover, certain mechanisms of resistance to cytotoxic chemotherapy for resected lung cancer have been studied recently.^17)^ It has been reported that adjuvant therapy with tegafur-uracil for the patients with lung cancer harboring EGFRmt is inferiorly effective than those without mutation because of its anti-apoptotic mechanism.^15,16)^ Our results were consistent with previous reports.^14,15)^ However, the clinical perspectives associated with the lesser effectiveness of adjuvant platinum doublet chemotherapy have not been presented in any of those studies.

The difference of pathological patterns may be related to the efficacy of the adjuvant chemotherapy between the EGFRmt and EGFRwt groups. In this study, the distribution of pathological stages between the two groups was not significantly different. Therefore, we had expected that the RFS and OS would also be similar. However, the EGFRmt group had a significantly worse RFS than the EGFRwt group. Furthermore, multivariate analysis revealed that the EGFR status was a significant risk factor for recurrence, and that lymphatic invasion was also an independent factor which was not statistically related to the EGFR status. Compared with the EGFRwt group, in the EGFRmt group, there was a higher percentage of lymph node metastasis as the main style of cancer progression, papillary pattern predominance, and micropapillary pattern presence.^18,19)^ However, EGFRmt lung cancer has been linked to better surgical outcome than EGFRwt lung cancer.^20–23)^ It is well established that the lepidic histological subtype is the major component of lung adenocarcinomas harboring EGFR mutations. This is the low-grade subtype and rarely associated with lymph node metastasis.^19,23)^ In these big data studies, cases of lung cancer with EGFRmt were characterized by an earlier stage, part-solid ground-glass opacity (low consolidation tumor ratio), and a lower frequency of lymphatic permeation and vessel invasion.^20,24,25)^ According to a previous report, the presence of a pure-solid nodule in lung cancer with EGFRmt is associated with dismal prognosis even in clinical stage I disease.^26)^ Our study implied a bimodal distribution of histological patterns in lung cancer with EGFRmt; early-stage disease is characterized by a more lepidic component, while advanced-stage disease involves micropapillary histological components. Hypothetically, lung adenocarcinoma with EGFRmt may develop through a unique carcinogenic pathway in which the low-grade lepidic subtype progresses to the high-grade micropapillary subtype.^27)^ Therefore, we should recognize the difference between early-stage and locally advanced-stage in lung cancer with EGFRmt. The causal relationship between EGFRmt and the histological subtype is an important subject in translational research. Prospective randomized controlled studies may be necessary to obtain a precise understanding of the clinicopathological characteristics of lung cancer harboring EGFRmt.

The selection of a local intervention, such as surgery, and radiotherapy (including chemoradiotherapy), or EGFR-TKI prescription in patients with lung cancers harboring EGFRmt following postoperative recurrence remains under debate. In this study, the OS of patients in the EGFRmt group was comparatively worse than that reported in other clinical trials.^10)^ A delay in the administration of EGFR-TKI might lead to the worse prognosis. Nevertheless, we should be careful regarding the inclusion of patients in the real-world setting of this study and selection bias of previous clinical trial data. Previous retrospective studies have shown the survival benefit of EGFR-TKI therapy at the time of recurrence.^21,28,29)^ The ADAURA trial clearly showed the advantage of early administration of osimertinib.^9,10)^ Immediate postoperative driver gene treatment may be important.

The results of our study raised the following clinical questions. The first question pertains to the necessity of adjuvant platinum-doublet therapy before adjuvant therapy with osimertinib.^13)^ Subgroup analysis of the ADAURA trial revealed that adjuvant treatment of osimertinib was better with and without adjuvant platinum-doublet treatment. However, there was no comparison conducted between patients with and without adjuvant chemotherapy before therapy with osimertinib.^30)^ In case platinum-doublet therapy is ineffective in patients with EGFRmt, upfront therapy with osimertinib should be selected. The second question pertains to the lack of value of pathological factors as determinant factors of perioperative treatment. In the adjuvant setting, high-grade histological patterns were not identified as independent risk factors by this study. At present, there are a few studies investigating the risk factors of preoperative intervention cases including pathological information (e.g. histological subtype and lymphovascular invasions).^19)^ Pathological findings may be considered for the selection of lung cancer standard therapy in perioperative situations.

### Limitations

This study had limitations due to a single-center retrospective design. The total number was limited, and the patient’ background was not adjusted. In addition, the implementation of adjuvant treatment possibly depended on the preference of doctors and patients. Moreover, the treatment guideline and first-line medicine for lung cancer changed during the observation period. In this study, the methods used for EGFR testing were changed during observation period from the cobas EGFR Mutation Test v2 (Roche Molecular Systems, Pleasanton, CA, USA) to the Oncomine Dx Target Test (Thermo Fisher Scientific, Waltham, MA, USA).

## Conclusion

Conventional cisplatin-based adjuvant chemotherapy may be less effective for the patients with lung cancer harboring EGFRmt who underwent complete resection versus those without mutation. EGFR status was identified as an independent risk factor for recurrence. Differences in the style of progression and histological pattern related to EGFRmt may be associated with the efficacy of adjuvant chemotherapy.

## Declarations

### Ethics approval and consent to participate

This retrospective cohort study used data from Kyoto University Hospital (Kyoto, Japan) and was approved by the Institutional Review Board of Kyoto University Hospital (approval number: R3829). The patients had the option to opt out from the study. Thus, the requirement for written informed consent was waived.

### Funding

None declared.

### Conflicts of interest/Competing interests

Hiroshi Date received lecture fees from Astrazeneca and Johnson&Johnson. The others had no conflict of interest.

### Data availability

The data that support the findings of this study are available from the corresponding author upon reasonable request.

### Authors contributions

TK collected and analyzed the data, visualized the figures, and wrote the original draft.

TM, SN, and HD reviewed and edited the draft as supervisor.

KT and AY reviewed pathological findings and supervised the draft.

All authors read and approved the final manuscript.
